# Adipose tissue-derived mesenchymal stem cells differentiated into hepatocyte-like cells *in vivo* and *in vitro*

**DOI:** 10.3892/mmr.2014.2935

**Published:** 2014-11-13

**Authors:** LIBO YIN, YUHUA ZHU, JIANGANG YANG, YIJIANG NI, ZHAO ZHOU, YU CHEN, LIXING WEN

**Affiliations:** Department of Traumatic Surgery, Changzhou No. 2 People’s Hospital, Nanjing Medical University, Changzhou, Jiangsu 213000, P.R. China

**Keywords:** differentiation, adipose tissue, mesenchymal stem cells, liver injury, cell

## Abstract

Cell-based therapy is a potential alternative to liver transplantation. The goal of the present study was to examine the *in vivo* and *in vitro* hepatic differentiation potential of adipose tissue-derived mesenchymal stem cells (AT-MSCs) and to explore its therapeutic use. AT-MSCs were isolated and cultured with hepatic differentiation medium. Bioactivity assays were used to study the properties of AT-MSCs. The morphology of differentiated AT-MSCs in serum-free hepatic differentiation medium changed into polygonal epithelial cells, while the morphology of AT-MSCs in a similar medium containing 2% fetal bovine serum remained unchanged. The differentiated cells cultured without serum showed hepatocyte-like cell morphology and hepatocyte-specific markers, including albumin (ALB) and α-fetoprotein. The bioactivity assays revealed that hepatocyte-like cells could take up low-density lipoprotein (LDL) and store glycogen. Furthermore, trichostatin A (TSA) enhanced ALB production and LDL uptake by the hepatocyte-like cells, analogous to the functions of human liver cells. ALB was detected in the livers of the CCl_4_-injured mice one month post-transplantation. This suggested that transplantation of the human AT-MSCs could relieve the impairment of acute CCl_4_-injured livers in nude mice. This therefore implied that adipose tissue was a source of multipotent stem cells which had the potential to differentiate into mature, transplantable hepatocyte-like cells *in vivo* and *in vitro*. In addition, the present study determined that TSA was essential to promoting differentiation of human MSC towards functional hepatocyte-like cells. The relief of liver injury following treatment with AT-MSCs suggested their potential as a novel therapeutic method for liver disorders or injury.

## Introduction

Certain liver diseases lead to hepatocyte damage that can progress to liver failure, for which a transplant may be the patients’ only treatment option. However, transplant organs are a highly limited resource, and transplant rejection remains to be an unresolved problem. The replacement of hepatocytes by stem cells or stem cell-stimulated endogenous and exogenous regeneration are the major goals of liver-directed cell therapy (1–4). Mature proliferating hepatocytes have the potential to be used as hepatic cell replacement (5). However, hepatocyte progenitors are still required under certain circumstances, such as the impaired ability of differentiated hepatocytes to further divide (6). Studies have suggested that reservoirs of stem cells may reside in organ tissues in order to promote self-repair and regeneration (7). However, adult stem cells have been suggested to be more plastic than once believed (8). Adult stem cells can be isolated from human lipoaspirates and differentiate toward osteogenic, adipogenic, neurogenic, myogenic and chondrogenic lineages (9). While stem cells derived from different tissues have shown promise for therapeutic applications, Lagasse *et al* (10) demonstrated that transplanted purified hematopoietic stem cells could give rise to hepatocytes and restore liver function in fumarylacetoacetate hydrolase-deficient mice. In humans, female recipients of male bone marrow (BM) were found to have hepatocytes that contained the Y chromosome (11), implying that hepatocytes could be derived from BM cells (12). Several studies have indicated that transplanted BM cells adopt the phenotype of hepatocytes and restore liver function by cell fusion rather than differentiation (13,14). Kern *et al* (3) and Wagner *et al (*15) compared mesenchymal stem cells (MSCs) derived from human adipose and bone marrow with respect to morphology, the success rate of isolating MSCs, colony frequency, expansion potential, multiple differentiation capacity and immune phenotype. They showed that adipose tissue-derived mesenchymal stem cells (AT-MSCs) had similar characteristics to those of BM mesenchymal stem cells (BMSCs). Adipose tissue contains stem cells similar to BMSCs; these cells could be isolated from cosmetic liposuctions and grown easily under standard tissue culture conditions (3). The multi-lineage differentiation capacity of AT-MSCs cells has been confirmed (3,9,15).

The aim of the present study was to investigate whether AT-MSCs had the potential to differentiate into functional hepatocytes *in vitro* and *in vivo*, in order to provide a novel stem cell therapy for the treatment of liver diseases.

## Materials and methods

All procedures were performed according to manufacturer’s instruction unless noted otherwise.

### Isolation and culture of MSCs from adipose tissue

Human adipose tissue was collected following liposuction surgery performed at Zhongshan Hospital affiliated to Xiamen University (Xiamen, China). Written informed consent and approval were obtained from the patients or their family, and the Human Research Ethical Committee of Zhongshan Hospital Xiamen University (Xiamen, China). The lipoaspirates (10–20 ml) were washed twice with an equal volume of phosphate-buffered saline (PBS; Digestive Diseases Institute of Xiamen University, Xiamen, China) and digested with 0.075% collagenase I (Sigma, St. Louis, MO, USA). Red Blood Cell Lysis Buffer (Beyotime, Haimen, China) was used to eliminate erythrocytes; cells were passed through a 40-μm mesh filter, and suspended in low-glucose Dulbecco’s modified Eagle’s medium (DMEM-LG; Gibco-BLR, Carlsbad, CA, USA) supplemented with 10% fetal bovine serum (FBS) and 100 U/ml penicillin/streptomycin (Gibco-BLR). The resuspended stromal vascular fraction (SVF) cells were plated at a density of 5×10^5^/cm^2^ in a 100-mm culture dish (Corning; Corning, NY, USA). The fibroblastoid adherent cells were designated AT-MSCs. Cells were harvested at 90% confluence using Trypsin-EDTA (Gibco-BLR), and designated as passage 0 (P0). Cells at P3–P5 were used for the subsequent experiments unless otherwise stated.

### Growth kinetics of AT-MSCs

To determine the growth kinetics of cultured AT-MSCs, 60-mm culture dishes were seeded with 1×10^5^ cultured AT-MSCs (P3–P5). At several time-points (between days 2 and 12) following seeding, cells from duplicate dishes were harvested and counted. AT-MSC numbers were plotted against the number of days in culture, and the exponential growth phase of the cells was determined.

### Measurement of AT-MSC proliferation

For the cell proliferation assay, 2×10^3^ viable AT-MSC were seeded in each triplicate well in a 96-well plate. AT-MSCs proliferation was measured using a cell counting kit 8 (CCK-8; Beyotime). The plates were placed in a humidified incubator until the cells adhered to the plate; then 10 μl of the CCK-8 solution was added to each well and plates were incubated for another 2 h at 37°C prior to reading the absorbance at 450 nm using a microplate reader (550; Bio-rad Laboratories, Inc., Shanghai, China). The assay was repeated every day at the same time for one week.

### Cell cycle analysis

AT-MSCs in the exponential growth phase were detached with 0.25% trypsin-EDTA (Gibco-BRL), and the resultant pelleted AT-MSCs (10^6^) were gently suspended in 1 ml 70% ethanol (Xiamen Chemical Company, Xiamen, China) and kept for at least 6 h at −20°C. Cells were washed with PBS twice, incubated for 1 h at room temperature (RT) in the dark with 100 μl 2.5 mg/ml propidium iodide (PI) (Sigma-Aldrich, Shanghai, China) and 1 mg/ml RNase (Beyotime) in PBS. The cells were analyzed with a FACSscan flow cytometer (Beckman Coulter, Brea, CA, USA) with a 488 nm wavelength.

### Transmission electron microscopy

Cells at 80–90% confluence were harvested with 0.25% trypsin-EDTA and washed twice with cold PBS. Fixing solution (2.5% glutaraldehyde; Xiamen Xinlongda Chemicals Company, Xiamen, China) was added and the pellet was incubated for 4 h at 4°C. The cells were washed after 2 h (or overnight) at 4°C with 0.1 M PBS three times. The fixed samples, which could be stored stably for several months, were sent to the electron microscopy department in the School of Life Sciences at the Xiamen University for further processing and analysis using the JEM-2100HC transmission electron microscope (JEOL, Tokyo, Japan).

### Flow cytometry

AT-MSCs (10^6^) were trypsinized and incubated with fluorescein isothiocyanate (FITC)-conjugated CD34, CD45, CD90, phycoerythrin (PE) -conjugated human leukocyte antigen (HLA)-DR, CD11b, CD29, CD105 (mouse, monoclonal, 1:200; eBioscience, San Diego, CA, USA) or PE-conjugated CD3, CD73, CD117, (mouse, monoclonal, 1:200; BD Biosciences, San Jose, CA, USA) antibodies for 30 minutes at RT, followed by three washes. Staining with unconjugated anti-programmed death ligand (PDL) 1 (rabbit, polyclonal, 1:100; BD Biosciences), FITC-conjugated goat anti-rabbit immunoglobulin G (IgG) antibody (Ab) (goat, polyclonal, 1:200; BD Biosciences) was used as a secondary antibody. The fluorescent labeled cells were analyzed on a FACSscan flow cytometer (Becton-Dickinson, Franklin Lakes, NJ, USA) using CELLQuest Pro software (Becton-Dickinson).

### Immunofluorescence assay (IFA)

Cultured cells were fixed with 4% paraformaldehyde (Sigma) in PBS for 5 minutes at RT and permeabilized with 0.3% Triton X-100 (Sigma) for 20 minutes. Cells were then incubated with blocking solution consisting of PBS and 5% FBS at RT for 30 minutes. For immunofluorescence staining, primary antibodies against albumin (ALB), (rat, polyclonal, 1:50; Santa Cruz Biotechnology, Inc., Santa Cruz, CA, USA), α-fetoprotein (AFP) (mouse, mono, 1:50; Wuhan Boster Biological Technology, Ltd., Wuhan, China) and pan cytokeratin (panCK; mouse, mono, 1:50; Zhongshan Goldenbridge Biotechnology, Beijing, China) were used. Following incubation with the primary antibody, cells were incubated with FITC-labeled anti-rat, Dylight-conjugated anti-mouse or FITC-labeled anti-mouse (rabbit, polyclonal, 1:200; Jackson ImmunoResearch, West Grove, PA, USA) secondary antibodies for 1 h at 37°C and stained with DAPI (Sigma) to identify the cell nuclei. The cells were photographed with a structured illumination fluorescence microscope (DM IL LED; Leica Microsystems, Tokyo, Japan).

### Hepatic differentiation protocols

**Figure 1:**
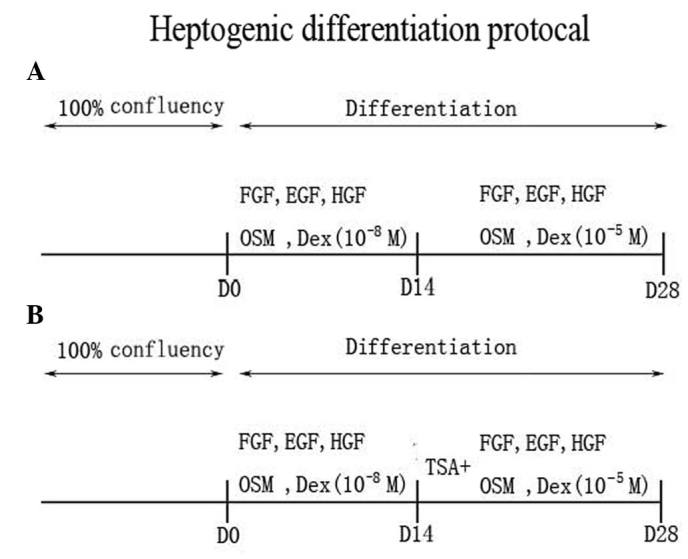
Schematic presentation of the hepatic differentiation protocols. All cells were pre-cultured in expansion medium until 100% confluence was achieved. (A) AT-MSCs were exposed to hepatic inducing agents at day 0; on day 14, dexamethasone concentration was changed from low (10^−8^ M) to high (10^−5^ M). (B) Protocol A with the addition of TSA on day 14. The media changed every two days; hepatic differentiation was then assessed at different time-points. F/E/HGF, fibroblast/epidermal/hepatocyte growth factor; D, day; OSM, oncostatin M; Dex, dexamethasone; TSA, trichostatin A.

To induce hepatogenic differentiation, AT-MSCs were plated after three passages on 5-mm culture dishes coated with collagen type I (Sigma) in expansion medium (DMEM-LG supplemented with 10% FBS). Following reaching confluence, cells were washed twice with PBS and cultured in basic hepatic differentiation medium supplemented with 1X insulin-transferrin-selenium (ITS; Sigma), 10^−8^ M dexamethasone (Sigma), 20 ng/ml epidermal growth factor (PeproTech EC, London, UK), 20 ng/ml fibroblast growth factor (FGF; PeproTech EC), 40 ng/ml oncostatin M (OSM; Sigma) and 40 ng/ml hepatocyte growth factor (HGF) (PeproTech EC). After 2 weeks, the medium was replaced with hepatic differentiation medium with an increased concentration of dexamethasone at 10^−5^ M (Fig. 1A) and/or 1 μM trichostatin A (TSA; Sigma) (Fig. 1B). The differentiation medium was used with or without serum. 2 ml of differentiation medium was added to each 12-well culture dish and changed twice a week. Hepatic differentiation was identified by cell morphology, immunohistochemistry, reverse transcription polymerase chain reaction (RT-PCR) analysis and biochemical functions at different time-points. All assays were performed with undifferentiated AT-MSCs as negative controls and human primary hepatocytes or HepG2 cells (Life and Science College, Xiamen University, Xiamen, China) as positive controls.

### In vitro adipogenic differentiation

The adipogenic differentiation assay was performed on AT-MSCs obtained between P3–P5 using the Human Mesenchymal Stem Adipogenic Differentiation Medium kit (Cyagen Biosciences, Guangzhou, China). Induction medium was used until the cells reached 100% confluence or post-confluence. Three days after confluence, the medium was changed to maintenance medium; 24 h later it was changed back to induction medium. Following completing three cycles of induction and maintenance, the cells were incubated for a further seven days in the adipogenic maintenance medium. The non-induced control cells were fed only with adipogenic maintenance medium. Adipogenic differentiation was confirmed by the formation of neutral lipid-vacuoles stainable with Oil Red O (Sigma). Non-induced cells were used as a control.

### In vitro osteogenic differentiation

Cells were treated with osteogenic medium (Osteogenic Differentiation kit; Cyagen Biosciences) for two weeks and the induction medium was changed every three days. Osteogenesis was assessed by von Kossa staining (Cyagen Biosciences). Non-induced cells were used as a control.

Total RNA was isolated from hepatic differentiated AT-MSCs (Trizol; Invitrogen, Carlsbad, CA, USA); 2 μg total RNA was used for reverse transcription (RevertAid First Strand cDNA Synthesis Kit; Fermentas, Burlington, Ontario, Canada). The cDNA was amplified using LaTaq (Takara BIO, Otsu, Shiga, Japan). Primers, synthetized by Sangon Biotech (Shanghai, China), were used to correspond with human gene sequences (Genbank database, www.ncbi.nlm.nih.gov/genbank): ALB sense, 5′-CCCCAAGTGTCAACTCCAA-3′ and antisense, 5′-AAAGCAGGTCTCCTTATCGT-3′; AFP sense, 5′-GGCTGACATTATTATCGGACAC-3′ and antisense, 5′-GTTCCTCTGTTATTTGTGGC-3′; and β-actin sense, 5′-TGAAGGTCGGAGTCAACGGATTTGGT-3′ and antisense, 5′-CAT GTGGGCCATGAGGTCCACCAC-3′ were used as an internal control for PCR. Amplification reactions were performed using an Eppendorf thermocycler (Mastercycler Nexus Gradient, Eppendorf, Germany) at 94°C for 30 seconds, 55°C for 30 seconds and 72°C for 60 seconds for 35 cycles. The PCR products were then separated by electrophoresis on 1.5% agarose gels (Sigma). The PCR sequencing product was confirmed by automatic sequencing (ABI 3730XL; Applied Biosystems, Foster City, CA, USA).

Periodic acid-Schiff (PAS) staining (Beyotime) was used for the detection of glycogen storage in hepatogenic differentiated cells. Cells were fixed with 10% formaldehyde (Beyotime) oxidized in 1% periodic acid (Beyotime) for 10 minutes and rinsed twice with water. Subsequently, cells were treated with Schiff’s reagent (Beyotime) for 10 minutes and then rinsed with water.

### Uptake of low-density lipoprotein

The uptake of lipoprotein was detected with the Dil-Ac-LDL staining kit (Biomedical Technologies, Stoughton, MA, USA).

### Animal model and cell transplantation

Animal experiments were conducted with permission from the Ethical Committee for Animal Experimentation of Xiamen University (Xiamen China) and according to P.R. China legislation. Immune-deficient BALB/c nude mice were obtained from the National Rodent Laboratory Animal Resources, (Shanghai, China); the animals were kept in animal house of Xiamen University according to internationally accepted principles. Animals were housed under standard laboratory conditions of light (12-h light/dark cycle) at 25±2°C, with a humidity of 55±5%, rats were fed a standard mice pellet diet and tap water *ad libitum*. All the mice were male. Mice were administered a single abdominal injection of olive oil containing 10% carbon tetrachloride (CCl_4_; Xiamen Chemical Company) at a dose of 100 μl/20 g body weight (bw). Cells for transplantation or control solutions were injected into the tail vein: Group A control mice were injected with 100 μl 10% CCl_4_/20 g bw only (n=9), group B received 100 μl PBS (n=9) and group C received 100 μl PBS containing AT-MSCs (5×10^5^ cells) (n=9). Following this procedure, three mice from each group were sacrificed on days one, three and seven. Blood samples were collected and whole livers were removed, fixed and prepared for further analysis. The serum concentrations of ammonia, alanine aminotransferase (ALT), aspartate aminotransferase (AST) and direct bilirubin (DBIL) were detected by histological analysis of liver tissue at days one, three and seven. To evaluate engraftment of AT-MSCs in the liver, two additional groups of mice were used: Group I mice were administered 100 μl/20 g bw of 10% CCl_4_, followed by AT-MSCs (5×10^5^ cells) after 24 h by injection into the tail vein (n=3); group II mice were injected with AT-MSCs (5×10^5^ cells) only (n=3). One month later, these mice were sacrificed, and the livers were removed and fixed for further study. Histological analysis of liver tissues was conducted by serial tissue sectioning and staining with hematoxylin and eosin (H&E; Beyotime) or immunohistochemical examination for human-specific ALB expression, as described above.

### Two-way mixed lymphocyte reaction (MLR) assay

The two-way MLR assay was used in order to detect whether lymphocyte aggregation was inhibited by AT-MSCs. The MLR was performed in 96-well microtiter plates using RPMI 1640 (Gibco-BRL) medium supplemented with 10% FBS. Purified T cells derived from two different donors were plated at 2×10^5^ cells per donor per well. Peripheral blood mononuclear cells (PBMCs) from two different donors were used as the ‘responder cells’ for the MLR. PBMCs were prepared by centrifugation of leukapheresed peripheral blood cells (Innovadyne, Santa Rosa, CA, USA). The derived lymphocytes were tested using flow cytometry with antibodies for CD3 and CD19 (BD Biosciences). T cells were mixed in complete culture medium at 2×10^5^ cells per donor per well in 96-well microtiter plates. Adipose tissue-derived cells were added to the MLR at 1, 250, 2,500, 5,000 or 10,000 cells per well. Stimulator cells were irradiated with 5,000 rads of γ-radiation with a cesium irradiator (XH BRI-1000; Baxter, Shanghai, China) prior to being added to the culture wells at the various concentrations. Control cultures consisted of T cells from two donors plated in medium alone (no stimulator cells). Triplicate cultures were set up for each condition. Cultures were pulsed with bromodeoxyuridine (BRDU; Millipore) on day 5 for 10 h. The optical density (OD) of the plates was then read with a spectrophotometer microplate reader (Biorad) set at a wavelength of 450 nm. All the above steps were performed at RT. The percentage of suppression was calculated using the following formula: Percentage suppression=(1-[(Test cell + MLR OD value) ÷ MLR OD value]) ×100%.

### Statistical analysis

Results are expressed as mean ± standard deviation. Statistical analyses were performed using least significant difference (LSD) t-test after one-way analysis of variance (ANOVA) or Student’s t-test. P<0.05 was considered to indicate a statistically significant difference between values.

## Results

### Characterization of AT-MSCs

#### Morphology and ultramicrostructure of AT-MSCs

**Figure 2:**
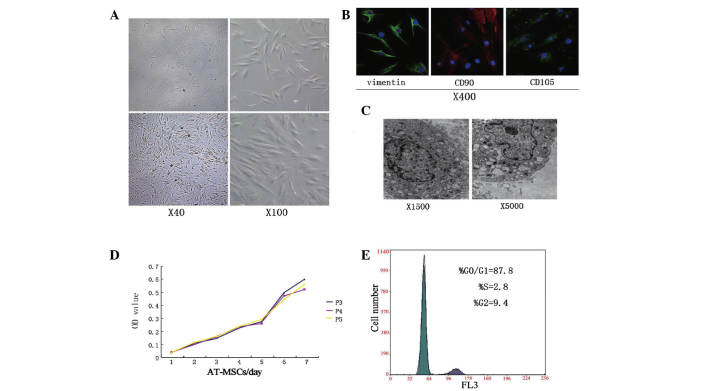
AT-MSC morphology. (A) AT-MSCs showed a fibroblast-like morphology, forming a CFU-F upon confluence. (B) Cells were stained for 1) vimentin and CD90 (FITC, green), 2) CD105 (Dylight, red), and 3) nuclei stained with DAPI. (C) Ultramicrostructure of AT-MSCs: Organelles had a naïve profile. (D) CCK-8 detection of growth kinetics; AT-MSCs of P3–5 had similar characteristics. (E) Cell cycle analysis showed that most cells were in dormant phase. CFU-F, colony forming unit fibroblast; FITC, fluorescein isothiocyanate; AT-MSCs, adipose tissue-derived mesenchymal stem cells; P, passage; OD, optical density.

AT-MSCs were cultivated from the mononuclear cell fraction of adipose tissue samples obtained from healthy donors. Cells were selected based on plastic adherence to ensure the removal of any contaminating hematopoietic cells, AT-MSCs expanded easily *in vitro* and exhibited a fibroblast-like morphology (Fig. 2A). The expression of mesenchymal stem cell markers, detected by immunofluorescence, was high in cultured AT-MSCs. The majority of cultured AT-MSCs expressed vimentin, and >90% highly expressed CD90 and CD105 (Fig. 2B). Following subsequent passages, differentiated cells displayed homogeneous morphologies and high rates of proliferation. Examination of AT-MSCs by electron microscopy displayed the presence of numerous surface microvilli. However, it also revealed a limited presence of organelles, including Golgi bodies, rough endoplasmic reticula, mitochondria; by contrast, the differentiated cells showed significant presence of organelles, including plate-like bodies (Fig. 2C).

### Cell cycle and growth patterns

AT-MSCs at P3–P5, showed a dynamic growth pattern, with duplication time of 3.00±0.28 days. In direct proliferation experiments, AT-MSCs of different passages (P3–P5) showed similar biological characteristics (Fig. 2D) and a stage of rapid cell proliferation approximately five days following cell culture (Fig. 2E). The patterns of proliferation as well as the cell cycle profiles demonstrated that these AT-MSCs displayed classical stem cell features.

### Phenotypic characterization of AT-MSC populations

**Figure 3:**
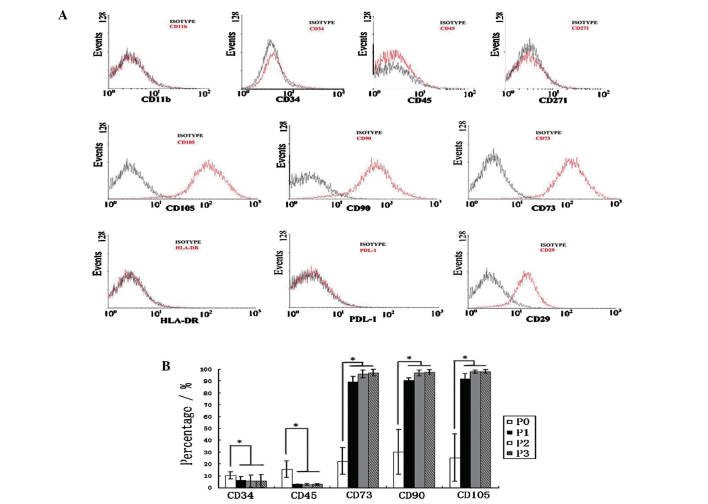
AT-MSCs express a unique set of CD markers. (A) Flow cytometric analysis of the expression of multiple CD antigens (red lines, representative histograms; black lines, respective isotype controls). (B) Changes in expression of CD markers at different passages. Values represent mean ± standard error of the mean (n=6). Data were analyzed by LSD-t-test following a one way analysis of varience. The expression of all markers shown at P0 differed significantly from that shown at P1/P2/P3 (P<0.05, LSD-t-test); with no difference in CD marker levels at P1, P2, P3 (P>0.05, LSD-t test). AT-MSCs, adipose tissue-derived mesenchymal stem cells; P, passage; HLA-DR, human leukocyte antigen-DR; PDL-1, programmed death-ligand 1; LSD, least significant difference.

Cell surface markers of AT-MSCs at P3–P5 were analyzed by flow cytometry. The average expression of the following markers from cells of all donors (n=6) were: CD11b (2±0.4%), HLA-DR (3.4±0.8%), PDL-1 (1.4±0.4%), CD29 (96±1.3%), CD34 (5.5±5.2%), CD45 (2.6±0.7%), CD73 (97±2.6%), CD90 (97.5±2%), CD105 (96.7±1.7%), CD271 (2.3±1.2%) (Fig. 3A). These results confirmed that the AT-MSCs expressed characteristic stem cell-associated surface markers CD29, CD73, CD90, CD105, while lacking expression of CD34, CD45, HLA-DR and PDL-1 (Fig. 3A). The hematopoietic lineage markers CD34, CD45 and other markers CD90, CD105 and CD73 were observed by flow cytometry in subsequent cultures of AT-MSCs. These markers were considered the minimum criteria for MSCs. Expression of the MSC markers was found to differ among passages. Of note, AT-MSCs of passage 0, AT-MSCs that were separated from human adipose tissue without cell culture, expressed higher CD34 and CD45 and lower CD73, CD90 and CD105. With increasing time of AT-MSCs in culture, hematopoietic lineage markers (CD34, CD45) were decreased, while expression of CD73, CD90 and CD105 intensified (Fig. 3B). Therefore, SVF in P0 expressed significantly different marker profiles from that of AT-MSCs at P1–P3 (P<0.05, one-way ANOVA and P<0.05, LSD-t-test).

### Multi-differentiation capacity of AT-MSCs

**Figure 4:**
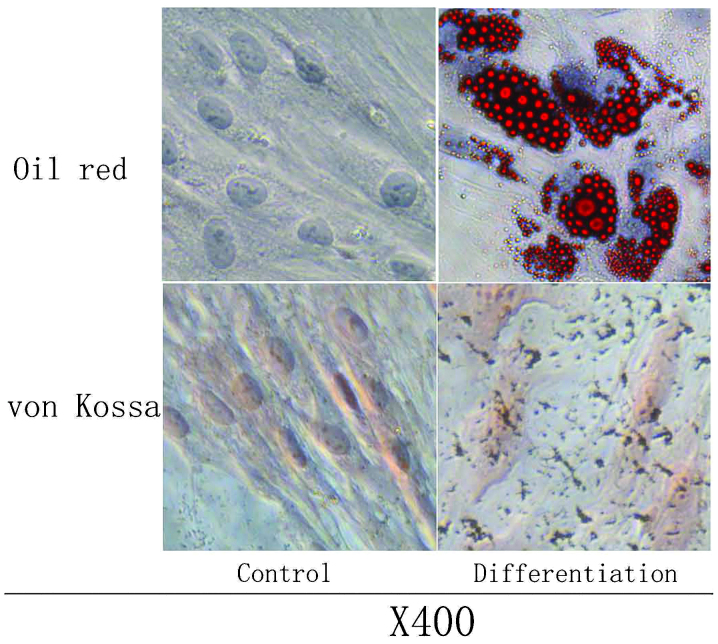
Multi-differentiation capacity of AT-MSCs. Evaluation of adipogenic differentiation of AT-MSCs: Oil Red O staining for the presence of intracytoplasmic lipid-rich droplets. Evaluation of osteogenic differentiation: Extracellular calcium mineralization following osteogenic differentiation stained black; nuclei stained with neural red (von Kossa). No staining observed in untreated AT-MSCs for either condition. AT-MSCs, adipose tissue-derived mesenchymal stem cells.

The osteogenic and adipogenic potential of AT-MSCs was evaluated at P3–P5. The adipogenic potential was assessed by induction of post-confluent AT-MSCs. Vacuoles appeared after three days of induction, and a consistent cell vacuolation was evident in the cytoplasm. Vacuoles stained strongly for fatty acids with Oil O Red. The lipid vacuoles were identified as bright red inclusions within cells, while the nuclei were stained dark blue with DAPI. Evidence for osteogenic differentiation was observed as morphological changes, which appeared during the first week of subculture. At the end of the 21-day induction period, calcium crystals were clearly visible in culture, and cell differentiation was confirmed by von Kossa staining for calcium. Extracellular calcium mineralization following osteogenic differentiation was visualized as black stains, and the nuclei stained with Neutral Red appeared as pink (Fig. 4).

### Hepatic differentiation of AT-MSCs in vivo

#### Morphological changes in cultured AT-MSCs

**Figure 5:**
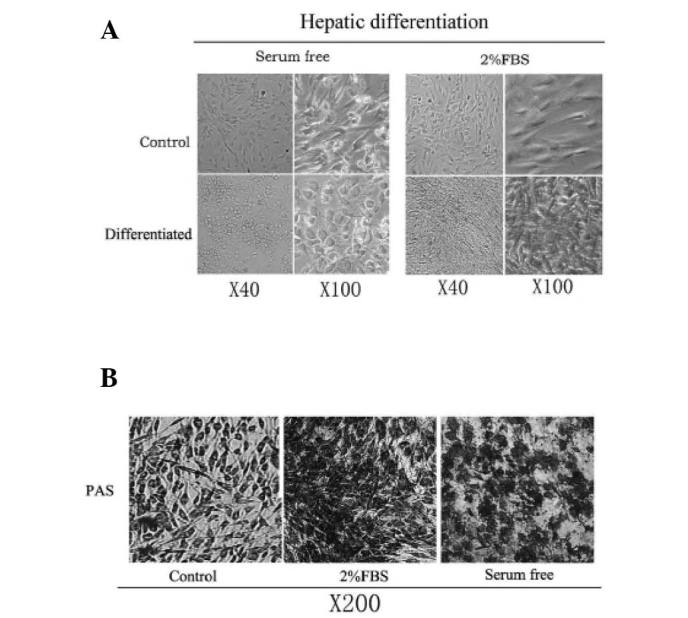
Hepatic differentiation of human AT-MSCs cultured in differentiation medium with or without serum for 14 days. (A) Glycogen storage ability was detected by PAS staining. (B) Morphological changes (polygonal and round shapes) in cells grown in medium without serum; no change in cells grown in medium plus 2% FBS. AT-MSCs, adipose tissue-derived mesenchymal stem cells; PAS, periodic acid-Schiff stain; FBS, fetal bovine serum.

Protocols shown in Fig. 1A and B were used to detect whether FBS could influence the hepatic induction procedure, in particular the morphological changes of the cells. Differentiation medium with or without 2% FBS was added to confluent AT-MSCs. During the initiation step of hepatic differentiation, the cells treated with serum-free media gradually lost their fibroblastic morphology and developed a broader, flatter shape; these cells subsequently developed a polygonal shape during differentiation (Fig. 5A). The contraction of the cytoplasm progressed further during maturation, and during differentiation the majority of treated cells became dense and round with clear or double nuclei. By contrast, hepatic differentiation medium with 2% FBS showed no significant morphological changes (Fig. 5A). The induced cells became more dense and elongated, both in the presence and absence of TSA. AT-MSCs were analyzed for glycogen-storage ability using PAS staining. As shown in (Fig. 5B), the serum-free medium-induced hepatocyte-like cells were strongly positive for PAS staining. However, cells with 2% FBS in the differentiation medium showed relatively weaker staining, and undifferentiated AT-MSCs were weakly positive for PAS staining. It was concluded that the serum-free induction medium was important for the differentiation of AT-MSCs into hepatocyte-like cells, as this dramatically changed the morphology of AT-MSCs from fibroblastic to epithelial.

### Function of hepatocyte-like cells derived from AT-MSCs

**Figure 6:**
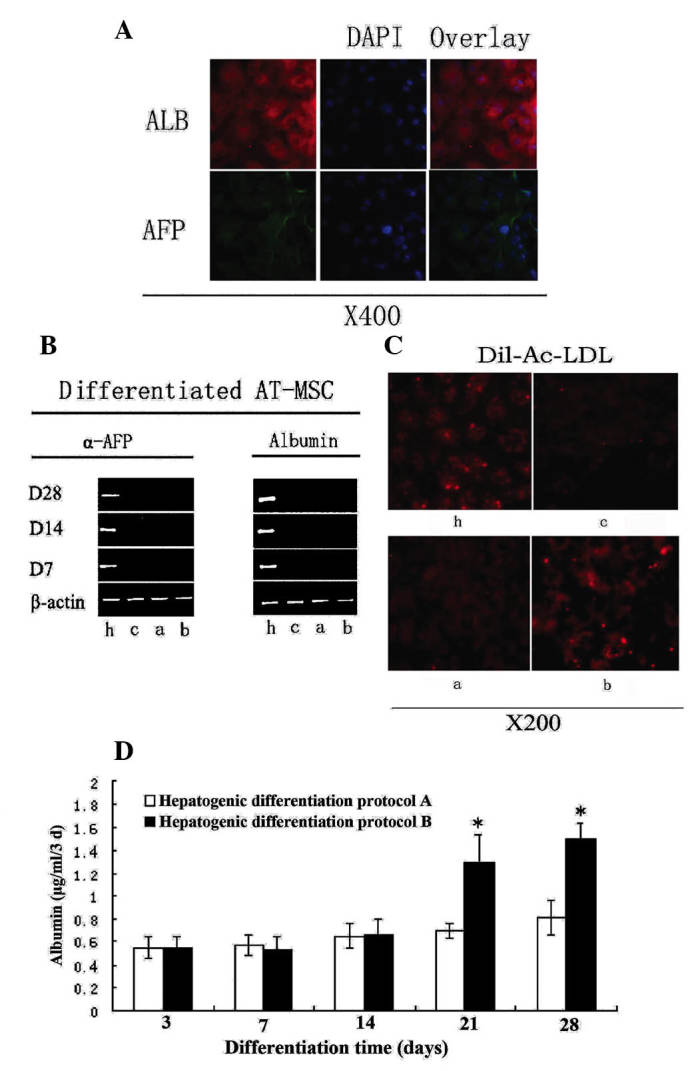
Differentiation potential of AT-MSCs exposed to hepatogenic differentiation medium. (A) ALB expression at day 14 and AFP expression at day 7. (B) Reverse transcription-polymerase chain reaction showed expression levels of ALB and AFP (using β-actin as a control). (C) AT-MSCs-derived hepatocyte-like cells were analyzed for LDL-uptake at day 28. (D) ALB levels secreted by AT-MSCs-derived hepatocyte-like cells. Data are presented as mean ± standard deviation and analyzed by Student’s t-test (n=3), ^*^P<0.05 protocol A vs. protocol B. h, HepG2; c, control; a, hepatogenic differentiation protocol A; b, hepatogenic differentiation protocol B; AT-MSC, adipose tissue mesenchymal stem cells; ALB, albumin; AFP, α-fetoprotein; LDL, low density lipoprotein; Dil-Ac-LDL, dioctadecyl-tetramethyl-indocarbocyanine perchlorate acetylated LDL.

Assays were performed to investigate the functional competence of AT-MSCs-derived hepatocyte-like cells without serum. At day 14, hepatocyte-like cells expressed both ALB and AFP, as detected by immunostaining using anti-human specific antibodies (Fig. 6A). ALB is a hepatocyte-specific marker of mature functional hepatocytes, while AFP is a marker for immature hepatocytes. Hepatocyte-like cells also showed an ability to store glycogen (Fig. 5B). Furthermore, following 2 weeks of induction, LDL uptake was observed in the hepatocyte-like cells, but did not occur in untreated cells (Fig. 6C). LDL is a lipoprotein that carries cholesterol in hepatocytes. During maturation (28 days), the majority of induced hepatocyte-like cells became competent for LDL uptake, a phenotype that was enhanced by addition of TSA to the induction medium (Fig. 6C).

### TSA can boost the functions of hepatocyte-like cells derived from AT-MSCs

In an attempt to enhance the *in vitro* differentiation of AT-MSCs, TSA, a selective and reversible histone deacetylase inhibitor (16), was added to the culture media following exposure of cells to hepatogenic factors over 14 days of incubation. Treatment of undifferentiated AT-MSCs with increased concentrations of TSA resulted in wide-spread cell death and cell detachment (16); therefore, 1.5 μM TSA was used in experiments for the present study. Total RNA was isolated at 7, 14 and 28 days post-differentiation of AT-MSCs into hepatic lineage, and the expression of several hepatic genes was examined by RT-PCR. Undifferentiated cells were used as negative controls and HepG2 was used as the positive control. The expression pattern of differentiated AT-MSCs from protocol B (Fig. 1B) was used to compare the levels of the ALB and AFP genes relative to human β-actin at different time-points by RT-PCR. ALB expression was significantly enhanced by induction of differentiation. As AT-MSCs differentiated into hepatocyte-like cells, matured and became more functional, AFP expression gradually diminished, more notably when TSA was added from day 14 onwards (Fig. 6C).

In order to further assess the effect of TSA on differentiated cellular function, the capacity of LDL uptake of hepatocyte-like cells from protocol B was evaluated (Fig. 1B). TSA enhanced the LDL uptake capacity dramatically. When different cell types were co-cultured with LDL for 8 h, the signal indicating LDL uptake in the hepatocyte-like cells in the TSA induction medium was considerably brighter in comparison with cells in the basic induction medium. The negative control cells (undifferentiated AT-MSCs) were considerably darker than the induced cells treated according to protocol A or B (Fig. 6C).

TSA, when added exclusively to AT-MSCs at 100% confluence and without pre-treatment with hepatogenic medium, was not effective in stimulating mesenchymal-to-hepatic transition. However, AT-MSCs treated with TSA from day 14 onwards exhibited significantly upregulated ALB secretion rates (P<0.05, Student’s t-test) when compared with cells in basic differentiation cultures (Fig. 6D).

### Transplantation of AT-MSCs into CCl_4_-injured nude mice results in the improvement of liver function

**Figure 7:**
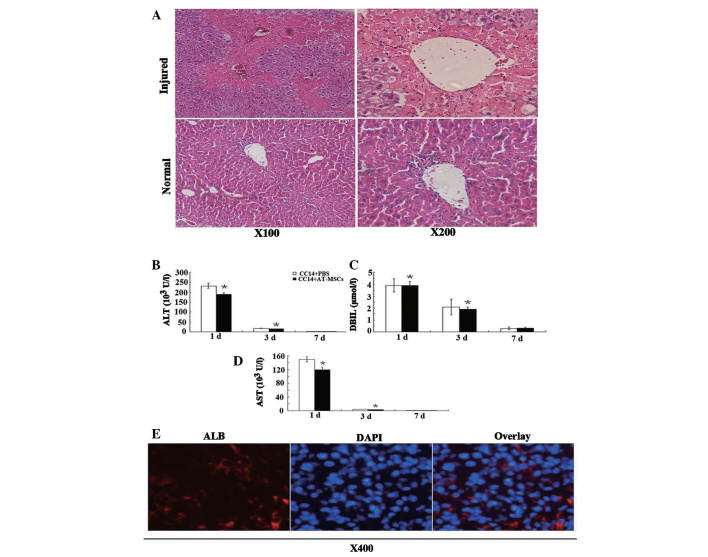
Therapeutic effect of AT-MSC administration. (A) One day following a single injection of 10% CCl_4_ (100 μl/20 g body weight) into the tail vein of nude mice, H&E staining showed pathological changes in liver cells, i.e., heavily damaged central veins of the liver and the morphological characteristics for hepatocytes vanished; (B–D) ALT, AST and DBIL levels decreased dramatically following AT-MSCs administration. Data are presented as mean ± standard deviation; analyzed by Student’s t-test, n=3, ^*^P<0.05 control vs. AT-MSC-treated group; (E) Human ALB detection in the livers of nude mice one month following injection of 5×10^5^ AT-MSCs into CCl_4_-injured mice. AT-MSC, adipose tissue mesenchymal stem cells; H&E, hematoxylin and eosin; ALT, alanine aminotransferase; AST, aspartate aminotransferase; DBIL, direct bilirubin; ALB, albumin.

Transaminase activity and direct bilirubin levels were measured at selected time-points to examine liver function following a single injection of CCl_4_. Transaminase activity peaked one day post-CCl_4_ injection, and pathological examination of H&E-stained sections revealed large areas of inflammation and hepatocyte denaturation (Fig. 7A). In comparison with normal liver tissue, both transaminase activity and direct bilirubin levels returned to normal seven days following the single injection of CCl_4_. Transaminase activity corresponded with H&E staining of liver pathology, while the serum ALT, DBIL and AST levels in the CCl_4_/PBS groups were significantly higher than those in the CCl_4_/AT-MSCs groups (Fig. 7B–D) (P<0.05, Student’s t-test). The therapeutic effects of AT-MSCs transplanted into mice with CCl_4_-induced acute liver injury were demonstrated by the decrease in serum ALT, AST activity and DBIL levels. Although differences in pathologies between CCl_4_/PBS and CCl_4_/AT-MSCs groups were not found, the results of the present study showed that transplantation of AT-MSCs had a beneficial effect on liver function *in vivo*.

### AT-MSCs reside in CCl_4_ injured livers and express liver-specific markers

Liver sections were examined by histochemical immunofluorescence with human ALB-specific antibodies (Fig. 7E), which demonstrated the incorporation of AT-MSCs into injured livers. Human ALB-positive cells were found in liver sections following injection of undifferentiated AT-MSCs; however, these cells did not exhibit the typical hepatocyte morphology (Fig. 7E). This indicated that AT-MSCs may have potential clinical application as treatment for acute liver injury.

### Low immunogenicity of the AT-MSCs

**Figure 8:**
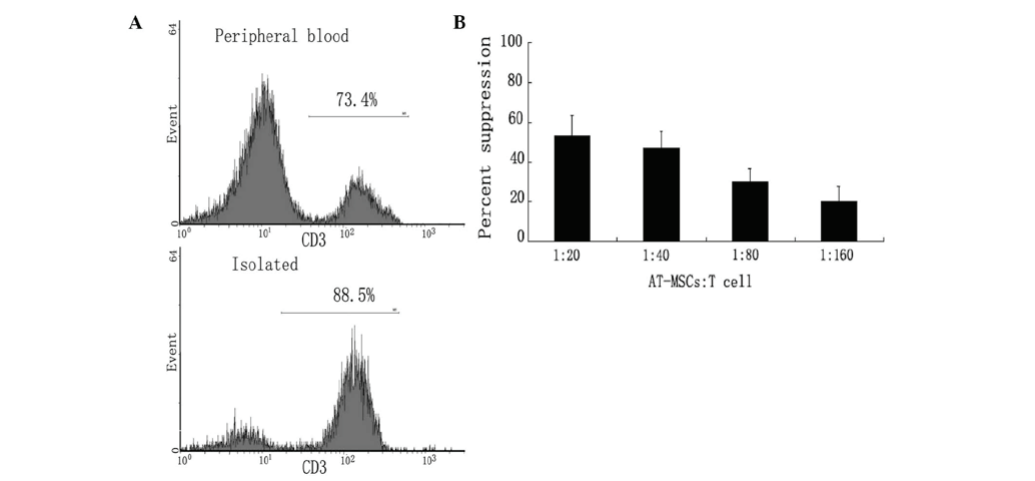
Immune suppressive capacity of AT-MSCs. (A) Ratio of T cells to total lymphocytes increased from 73.4% in peripheral blood to 88.5% following isolation. (B) Human AT-MSCs suppressed two-way MLR reactions; the immunosuppressive capacity was dose-dependent. AT-MSC, adipose tissue mesenchymal stem cells; MLR, mixed lymphocyte reaction.

The potential clinical use of AT-MSCs is attractive due to their low immunogenicity. Both activated and inactivated AT-MSCs lacked expression of co-stimulatory molecules, including CD40, CD54, CD80, CD86, or HLA-DR and HLA-ABC (data not shown). The inability of AT-MSCs to stimulate a T-cell response may be due to an inherently low immunogenicity status, or active immunosuppressive mechanisms mediated by the AT-MSCs. MLR assays with allogeneic T cells were performed to determine whether the fat-derived cells were immunosuppressive. The ratio of T cells to total lymphocytes increased from 73.4% in peripheral blood to 88.5% following isolation (Fig. 8A). AT-MSCs suppressed MLR cultures in a dose-dependent manner (Fig. 8B); T-cell aggregation was decreased compared with that of with that of controls (data not shown). In addition, AT-MSCs significantly suppressed T-cell proliferation in MLR. These data indicated that allogeneic AT-MSCs may be a potential source of cells for tissue repair or replacement. The present study also has important implications with respect to the ready availability of adult stem cells for clinical applications, and to the practical and commercial aspects of their manufacture and quality assurance. AT-MSCs are not only inherently non-immunogenic and have the ability to suppress proliferation of alloantigen- or mitogen-stimulated T cells, but they can also suppress immunoglobulin production by mitogen-stimulated B cells (27). The low immunogenicity and immunosuppressive characteristics show that human adipose tissue may be an ideal source for cell transplantation (27–29).

## Discussion

AT-MSCs have great potential for clinical applications in regenerative medicine. Transplantation of AT-MSCs may provide an easier, more efficient and safer method for the treatment of patients with liver disease than whole organ transplantation (17). Adipose tissue can be obtained in large quantities with minimally invasive procedures and AT-MSCs can be easily isolated and cultured *in vitro* (17–19). AT-MSCs have a broader differentiation potential than previously anticipated and can differentiate into all mesodermal lineage cells, including osteocytes, adipocytes and chondrocytes (20,21). AT-MSCs have characteristics similar to those of BMSCs; AT-MSCs have the ability to differentiate into osteogenic and adipogenic cells *in vitro*; however they do not express hematopoietic cell antigens. The mechanism of MSC differentiation into hepatocyte-like cells *in vitro* has not been determined extensively: Baertschiger *et al* (22) reported that co-culturing with Huh-7 cells was sufficient to induce hepatic differentiation. Chien *et al* (23) found that coating culture plates with different proteins affected human placenta-derived multi-potent cells, which when cultured in differentiation medium on poly-L-lysine-coated plates, changed morphology and became polygonal in shape. These morphological changes were less obvious when cells were cultured on fibronectin-coated plates.

TSA is a trigger for the further differentiation of human MSCs towards endodermal lineages (24). High levels of TSA can induce cell apoptosis and promote differentiation by inducing cell cycle arrest during the G0/G1 and G1/S phases (25). Vinken *et al* (25) reported that TSA counteracted the loss of liver-specific functions in primary rat hepatocytes (26) and enhanced intercellular communication through gap junctions. In the present study, TSA significantly boosted the function of hepatocyte-like cells derived from AT-MSCs, regardless of increases in the ALB mRNA and protein levels, in addition to enhancing their glycogen storage abilities.

*In vivo*, the transplanted cells were exposed to a dynamic host microenvironment laden with soluble mediators and immunoreactive cells. The present study hypothesized that at least three ubiquitous microenvironmental factors may affect the differentiation and function of the MSCs. Interleukin-1α (IL-1α) is a key regulator of hematopoiesis and the inflammatory process (30). Recent studies on mice have shown that MSCs have an inherent ability to counteract the deleterious inflammatory effects of IL-1α in injured tissues (31) In addition, tumor necrosis factor α, which has been shown to increase chemotaxis of MSCs, would also be clinically important if the efficiency of concentrating MSCs to the site of tissue injury was improved (32). Furthermore, stromal cell-derived factor-1α is a promoter of non-specific MSC migration (33,34). Therefore, co-therapy with a pharmacological agent, such as a cytokine receptor antagonist, may negate the deleterious effects of the microenvironment and optimize the therapeutic potential of MSCs (31,35). Human BMSCs used for transplantation have been expanded without significant loss of their differentiation capacities. Following transplantation into unconditioned adult mice, BMSCs not only migrated to the bone marrow but also into other tissues. Impairment of the tissue was shown to cause increased BMSC implantation not only in bone marrow and muscle but it was also shown to lead to further engraftment in the brain, heart and liver (36).

Oyagi *et al* (37) reported that BMSCs reduced hepatocyte apoptosis and promoted cell proliferation. In addition, they showed that autologous MSC transplantation prolonged the survival of dogs (38) and swine (39) receiving living donor liver transplantation. The present study clearly demonstrated that AT-MSCs can ease mouse acute hepatic injury *in vivo* by decreasing inflammation and apoptosis as well as increasing proliferation and recovery (as evidenced by decreased ALT, AST and DBIL levels). It was demonstrated that AT-MSCs are a novel source of cells that may be used to treat hepatic injury and/or dysfunction. The mechanisms by which hepatic differentiation occurs *in vivo* and hepatic function is restored, however, are still not fully elucidated. Oyagi *et al* (37) reported that BMSCs secreted HGF and suppressed inflammation when transplanted into CCl_4_-injured rats. HGF had the capacity to induce hepatic differentiation and suppress hepatocyte death (40). Silva *et al* (41) reported that the transplantation of MSCs reduced fibrosis through the secretion of cytokines, in particular vascular endothelial growth factor. BMSCs have the capacity to differentiate into hepatocyte-like cells in response to growth factor stimulation (37). Results of the present study showed that AT-MSCs can only reside in CCl_4_-injured livers, suggesting that hepatic differentiation of AT-MSCs may be induced by HGF and/or other cytokines secreted by the CCl_4_-injured livers. It remains elusive whether MSCs contribute to tissue repair by differentiation into tissue-specific cell types, or whether they produce trophic factors at the site of injury, which can stimulate tissue repair (42,43). MSCs are responsive to their environment, adapting function to local circumstances, and immunosuppressive properties appear to be induced under inflammatory conditions (43). Modification of the culture medium could modulate the properties of MSCs, for example, by enhancing immunosuppressive function and reducing susceptibility for lysis by cytotoxic T cells (44).

In conclusion, ALB was detected in the livers of the CCl_4_-injured mice one month post-transplantation. This suggested that transplantation of the human AT-MSCs could relieve the impairment of acute CCl_4_-injured livers in nude mice. This therefore implied that adipose tissue was a source of multipotent stem cells which had the potential to differentiate into mature, transplantable hepatocyte-like cells *in vivo* and *in vitro*. In addition, the present study determined that TSA was essential to promoting differentiation of human MSC towards functional hepatocyte-like cells. The relief of liver injury following treatment with AT-MSCs suggested their potential as a novel therapeutic method for liver disorders or injury. Human AT-MSCs may become a useful source for hepatocyte regeneration and may provide an alternative to liver transplantation.
